# Clinical Outcomes and New Treatments in Pediatric Brain Tumors

**DOI:** 10.3390/curroncol33090549

**Published:** 2026-09-10

**Authors:** Craig Erker, Sébastien Perreault

**Affiliations:** 1 Division of iHOPE, Department of Pediatrics, University of Alberta, Edmonton, AB T6G 2R3, Canada; craig.erker@ahs.ca; 2 Stollery Children’s Hospital, Edmonton, AB T6G 2B7, Canada; 3 Department of Neurosciences, University of Montreal, CHU Sainte-Justine, Montreal, QC H3T 1J4, Canada

## 1. Introduction

Central nervous system (CNS) tumors represent the most common solid tumors of childhood and remain the leading cause of cancer-related mortality among pediatric patients [1]. Although survival outcomes for many childhood malignancies have improved substantially over the past several decades, progress in pediatric neuro-oncology has been comparatively slower. Many survivors experience substantial long-term treatment-related morbidity including neurocognitive impairment, endocrine dysfunction, hearing loss, vascular complications, and psychosocial challenges [2]. These late effects underscore a central challenge in pediatric neuro-oncology: improving survival outcomes while minimizing treatment-associated toxicity and preserving long-term quality of life.

## 2. Overview of the Special Issue

This Special Issue of Current Oncology, entitled “Clinical Outcomes and New Treatments in Pediatric Brain Tumors,” brings together a series of manuscripts highlighting recent advances across the pediatric neuro-oncology field. The collection of articles addresses several key themes including advances in conventional treatment strategies, emerging targeted therapies, novel immunotherapeutic approaches, and efforts to reduce treatment-related toxicity.

Cheng et al. (Contribution 1) review the evolving role of chemotherapy in pediatric brain tumors and discuss how cytotoxic therapies continue to play a critical role in multimodal treatment strategies. Tsang and colleagues (Contribution 2) provide an overview of advances in radiation therapy, focusing on modern radiation techniques such as proton therapy that aim to maintain or improve disease control while reducing late toxicities.

Renzi et al. (Contribution 3) review targeted therapies for pediatric gliomas and NF1-associated tumors, highlighting the growing role of pathway-directed therapies such as MEK inhibitors. Complementing this work, Vairy and Michaiel (Contribution 4) examine the expanding landscape of small-molecule drugs in pediatric neuro-oncology, including emerging agents targeting molecular drivers such as the RAS/MAPK pathway and H3K27M mutations.

Rousseau and collaborators (Contribution 5) explore the potential role of tumor-treating fields in pediatric brain tumors, while Das and colleagues (Contribution 6) review the development of cellular, immunotherapy and oncolytic viral therapies. Finally, Coltin, Coleman, and Cacciotti (Contribution 7) discuss strategies aimed at reducing treatment-related toxicity and improving survivorship outcomes for children with CNS tumors. Schlosser and colleagues (Contribution 8) present a moving narrative describing the clinical journey of a child diagnosed with glioblastoma, told from both the physician and family perspectives. Together, these manuscripts provide a comprehensive overview of the rapidly evolving therapeutic landscape in pediatric neuro-oncology.

## 3. Chemotherapy and the Evolution of Systemic Therapy

Conventional chemotherapy has long served as a cornerstone of treatment for pediatric brain tumors. Cytotoxic chemotherapy has played a critical role not only in improving survival outcomes but also in enabling radiation-sparing approaches in young children whose developing brains are particularly vulnerable to the neurocognitive effects of cranial irradiation.

The role of chemotherapy continues to evolve as tumor biology becomes better understood. In medulloblastoma, molecular classification into WNT, SHH, Group 3, and Group 4 subtypes has enabled risk-adapted treatment approaches [3]. Current clinical trials are exploring therapy de-escalation strategies for favorable-risk WNT tumors while maintaining or intensifying therapy for higher-risk subgroups. These strategies reflect the broader goal of balancing oncologic control with reduction in long-term treatment toxicity.

## 4. Advances in Radiation Therapy

Radiation therapy remains an essential component of treatment for many pediatric CNS tumors including medulloblastoma and other embryonal tumors, ependymoma, and high-grade gliomas. Despite its effectiveness in controlling disease, radiation therapy is associated with substantial long-term complications such as neurocognitive decline, endocrine dysfunction, vasculopathy, and secondary malignancies [2]. As survival improves for children with brain tumors, minimizing these late effects has become a major focus of contemporary pediatric neuro-oncology.

Technological advances have significantly improved delivery of radiation therapy. Proton beam therapy has emerged as one of the most important developments in pediatric radiation oncology. Because of the Bragg peak phenomenon, proton therapy allows radiation dose deposition to occur directly within the tumor while minimizing exposure to surrounding normal tissues. Several studies suggest that proton therapy may reduce radiation exposure to critical brain structures and potentially decrease the risk of long-term neurocognitive and endocrine complications [4,5].

## 5. Targeted Therapies and Small Molecule Inhibitors

One of the most transformative developments in pediatric neuro-oncology has been the identification of molecular drivers underlying many childhood brain tumors. In pediatric low-grade gliomas, dysregulation of the RAS/MAPK signaling pathway is observed in the majority of tumors, most commonly through BRAF fusions, BRAF V600E mutations, or alterations associated with neurofibromatosis type 1 (NF1) [6].

These discoveries have led to the development of targeted therapies designed to inhibit key oncogenic signaling pathways. MEK inhibitors such as selumetinib have demonstrated clinical activity in children with recurrent or progressive pLGG [7]. Similarly, the combination of dabrafenib and trametinib has shown rapid and significant responses in tumors harboring BRAF V600E mutations. More recently, the pan-RAF inhibitor tovorafenib showed substantial activity in children with relapsed or refractory BRAF-altered pediatric low-grade gliomas in the FIREFLY-1 clinical trial [8]. Small-molecule agents targeting other molecular alterations are also under investigation. IDH inhibitors such as vorasidenib have recently shown improved progression-free survival in IDH-mutant gliomas [9].

## 6. Innovative Therapeutic Approaches

In parallel with the development of targeted therapies, several innovative treatment strategies are being explored to improve outcomes for children with brain tumors. Tumor-treating fields (TTFs) represent one such strategy. TTFs deliver alternating electric fields that disrupt tumor cell mitosis and inhibit tumor cell proliferation. Clinical trials in adult glioblastoma have demonstrated improved survival with the addition of TTFs to standard therapy [10], prompting interest in evaluating this technology in pediatric brain tumors.

## 7. Emerging Immunotherapies

Adoptive cellular therapies are also being investigated in pediatric neuro-oncology. CAR-T cell therapies targeting tumor antigens such as GD2 have demonstrated early signals of activity in children with diffuse midline gliomas and other aggressive CNS tumors [11]. Although these approaches remain in early stages of development, they represent an exciting frontier for future therapeutic innovation.

Immune checkpoint inhibitors (ICIs) have shown limited efficacy in most pediatric CNS tumors due to low tumor mutational burden and an immunosuppressive tumor microenvironment. In contrast, tumors with DNA mismatch repair deficiency (MMRd) demonstrate marked sensitivity to ICIs, driven by their ultra-hypermutated phenotype and highly inflamed microenvironment, with reported response rates up to 50–64% and 2-year survival reaching 43% [12,13]. Consequently, MMRd status has emerged as a key predictive biomarker for ICI response, while broader application of immunotherapy in pediatric neuro-oncology will require biomarker-driven strategies and combination approaches.

Another promising strategy involves oncolytic virotherapy. Oncolytic viruses selectively infect and destroy tumor cells while simultaneously stimulating antitumor immune responses. Early clinical studies evaluating oncolytic herpes simplex virus constructs in pediatric high-grade gliomas have demonstrated encouraging safety profiles and early biological activity [14].

## 8. Reducing Treatment-Related Toxicity and Survivorship

As survival outcomes improve for many pediatric CNS tumors, increasing attention has focused on reducing treatment-related toxicity and improving survivorship outcomes. Survivors of pediatric brain tumors frequently experience long-term complications affecting neurocognitive development, endocrine function, hearing, and psychosocial well-being [2]. Consequently, strategies aimed at mitigating treatment-associated toxicity are increasingly integrated into therapeutic decision-making.

Pharmacologic interventions have been explored to reduce treatment-associated toxicities. For example, sodium thiosulfate has demonstrated efficacy in reducing cisplatin-induced hearing loss in children receiving platinum-based chemotherapy [15]. Additional approaches include radiation dose reduction in biologically favorable tumors, use of proton therapy, and long-term survivorship programs designed to monitor and manage late effects.

## 9. Patient Perspectives

The experience of children suffering from CNS tumors and their impacted families provides an essential perspective that complements scientific and clinical advances. The article highlights the emotional, logistical, and medical challenges families face while navigating a devastating diagnosis for which therapeutic options remain limited. It emphasizes the importance of transparent communication, access to clinical trials, and the need for continued investment in pediatric brain tumor research. It also underscores how compassionate multidisciplinary care can meaningfully improve a child’s quality of life even in the setting of incurable disease. Importantly, the perspective of Ben’s family illustrates the determination of patients and families to contribute to research efforts that may benefit future children with similar diagnoses. By integrating both provider and family perspectives, this contribution reminds clinicians and researchers that progress in pediatric neuro-oncology must remain centered on the lived experiences and priorities of patients and their families.

## 10. Future Directions and Editorial Perspective

The rapid progress in pediatric neuro-oncology over the past decade reflects the growing integration of molecular biology, translational research, and collaborative clinical trials. Precision medicine approaches are increasingly guiding treatment decisions, and emerging therapies such as targeted inhibitors, immunotherapies, and gene-based strategies hold promise for improving outcomes in children with brain tumors.

Future advances will likely depend on several key priorities, including the development of biomarker-driven clinical trials, improved understanding of mechanisms of therapeutic resistance, and expansion of international collaborative research networks. In addition, novel diagnostic approaches such as liquid biopsy and advanced imaging techniques may improve disease monitoring and may facilitate earlier detection of relapse.

Ultimately, continued progress in pediatric neuro-oncology will require close collaboration between clinicians, researchers, patients, and advocacy organizations. The manuscripts presented in this Special Issue highlight both the remarkable advances achieved to date and the important challenges that remain. By integrating innovative therapies with strategies aimed at reducing toxicity, the field moves closer to the goal of improving both survival and quality of life for children diagnosed with CNS tumors.

## Data Availability

No new data were created or analyzed in this study. Data sharing is not applicable to this article.

## References

[1] Ostrom Q.T., Price M., Neff C., Cioffi G., A Waite K., Kruchko C., Barnholtz-Sloan J.S. (2022). CBTRUS Statistical Report: Primary Brain and Other Central Nervous System Tumors Diagnosed in the United States in 2015–2019. Neuro-Oncology.

[2] Armstrong G.T., Liu Q., Yasui Y., Huang S., Ness K.K., Leisenring W., Hudson M.M., Donaldson S.S., King A.A., Stovall M. (2009). Long-Term Outcomes Among Adult Survivors of Childhood Central Nervous System Malignancies in the Childhood Cancer Survivor Study. JNCI J. Natl. Cancer Inst..

[3] Northcott P.A., Buchhalter I., Morrissy A.S., Hovestadt V., Weischenfeldt J., Ehrenberger T., Gröbner S., Segura-Wang M., Zichner T., Rudneva V.A. (2017). The whole-genome landscape of medulloblastoma subtypes. Nature.

[4] Uemura S., Demizu Y., Hasegawa D., Fujikawa T., Inoue S., Nishimura A., Tojyo R., Nakamura S., Kozaki A., Saito A. (2022). The comparison of acute toxicities associated with craniospinal irradiation between photon beam therapy and proton beam therapy in children with brain tumors. Cancer Med..

[5] Aldrich K.D., Horne V.E., Bielamowicz K., Sonabend R.Y., Scheurer M.E., Paulino A.C., Mahajan A., Chintagumpala M., Okcu M.F., Brown A.L. (2021). Comparison of hypothyroidism, growth hormone deficiency, and adrenal insufficiency following proton and photon radiotherapy in children with medulloblastoma. J. Neuro-Oncol..

[6] Jones D.T.W., Gronych J., Lichter P., Witt O., Pfister S.M. (2011). MAPK pathway activation in pilocytic astrocytoma. Cell. Mol. Life Sci..

[7] Fangusaro J., Onar-Thomas A., Poussaint T.Y., Lensing S., Ligon A.H., Lindeman N., Banerjee A., Kilburn L.B., Lenzen A., Pillay-Smiley N. (2025). A phase 2 PBTC study of selumetinib for recurrent/progressive pediatric low-grade glioma: Strata 2, 5, and 6 with long-term outcomes on strata 1, 3, and 4. Neuro-Oncology.

[8] Kilburn L.B., Khuong-Quang D.-A., Hansford J.R., Landi D., van der Lugt J., Leary S.E.S., Driever P.H., Bailey S., Perreault S., McCowage G. (2023). The type II RAF inhibitor tovorafenib in relapsed/refractory pediatric low-grade glioma: The phase 2 FIREFLY-1 trial. Nat. Med..

[9] Mellinghoff I.K., Bent M.J.v.D., Blumenthal D.T., Touat M., Peters K.B., Clarke J., Mendez J., Yust-Katz S., Welsh L., Mason W.P. (2023). Vorasidenib in IDH1- or IDH2-Mutant Low-Grade Glioma. N. Engl. J. Med..

[10] Stupp R., Taillibert S., Kanner A., Read W., Steinberg D.M., Lhermitte B., Toms S., Idbaih A., Ahluwalia M.S., Fink K. (2017). Effect of Tumor-Treating Fields Plus Maintenance Temozolomide vs. Maintenance Temozolomide Alone on Survival in Patients with Glioblastoma: A Randomized Clinical Trial. JAMA.

[11] Monje M., Mahdi J., Majzner R., Yeom K.W., Schultz L.M., Richards R.M., Barsan V., Song K.-W., Kamens J., Baggott C. (2024). Intravenous and intracranial GD2-CAR T cells for H3K27M+ diffuse midline gliomas. Nature.

[12] Das A., Tabori U., Nahum L.C.S., Collins N.B., Deyell R., Dvir R., Faure-Conter C., Hassall T.E., Minturn J.E., Edwards M. (2023). Efficacy of Nivolumab in Pediatric Cancers with High Mutation Burden and Mismatch Repair Deficiency. Clin. Cancer Res..

[13] Das A., Sudhaman S., Morgenstern D., Coblentz A., Chung J., Stone S.C., Alsafwani N., Liu Z.A., Abu Al Karsaneh O., Soleimani S. (2022). Genomic predictors of response to PD-1 inhibition in children with germline DNA replication repair deficiency. Nat. Med..

[14] Kardani K., Gil J.S., Rabkin S.D. (2023). Oncolytic herpes simplex viruses for the treatment of glioma and targeting glioblastoma stem-like cells. Front. Cell. Infect. Microbiol..

[15] Brock P.R., Maibach R., Childs M., Rajput K., Roebuck D., Sullivan M., Laithier V., Ronghe M., Dall’Igna P., Hiyama E. (2018). Sodium Thiosulfate for Protection from Cisplatin-Induced Hearing Loss. N. Engl. J. Med..

